# Using a smartphone app to reduce cognitive vulnerability and mild depressive symptoms: Study protocol of an exploratory randomized controlled trial

**DOI:** 10.1186/s13063-016-1740-3

**Published:** 2016-12-28

**Authors:** Cezar Giosan, Cristina Mogoaşe, Oana Cobeanu, Aurora Szentágotai Tătar, Vlad Mureşan, Rareș Boian

**Affiliations:** 1 Department of Clinical Psychology and Psychotherapy, Babeș-Bolyai University, Cluj-Napoca, Romania; 2 Berkeley College, New York, NY USA; 3 Department of Psychology, University of Bucharest, Bucharest, Romania

**Keywords:** Depression, Prevention, Protocol, Randomized trial, CBT, Smartphone app

## Abstract

**Background:**

Depression is a major challenge worldwide, with significant increasing personal, economic, and societal costs. Although empirically supported treatments have been developed, they are not always available for patients in routine clinical care. Therefore, we need effective and widely accessible strategies to prevent the onset of the very first depressive symptoms. Mental health apps could prove a valuable solution for this desideratum. Although preliminary research has indicated that such apps can be useful in treating depression, no study has attempted to test their utility in preventing depressive symptoms. The aim of this exploratory study is to contrast the efficacy of a smartphone app in reducing cognitive vulnerability and mild depressive symptoms, as risk factors for the onset of depression, against a wait-list condition. More specifically, we aim to test an app designed to (1) decrease general cognitive vulnerability and (2) promote engagement in protective, adaptive activities, while (3) counteracting (through gamification and customization) the tendency of premature dropout from intervention.

**Methods/design:**

Romanian-speaking adults (18 years and older) with access to a computer and the Internet and who own a smartphone are included in the study. Two parallel randomized clinical trials are conducted: in the first one, 50 participants free of depressive symptoms (i.e., who obtain scores ≤4 on the Patient Health Questionnaire, PHQ-9) will be included, while in the second one 50 participants with minimal depressive symptoms (i.e., who obtain PHQ-9 scores between 5 and 9) will be included. Participants undergoing therapy, presenting with substance abuse problems, psychotic symptoms, and organic brain disorders, or serious legal or health issues that would prevent them from using the app, as well as participants reporting suicidal ideation are excluded. Participants randomized to the active intervention will autonomously use the smartphone app for 4 weeks, while the others will be given access to the app after 4 weeks from randomization. The primary outcomes are (1) cognitive vulnerability factors as defined within the cognitive behavioral therapy (CBT) paradigm (i.e., dysfunctional cognitions, irrational beliefs, and negative automatic thoughts) (for the first trial), and (2) level of depressive symptomatology (for the second trial). The app includes self-help materials and exercises based on CBT for depression, presented in a tailored manner and incorporating gamification elements aimed at boosting motivation to use the app.

**Discussion:**

This study protocol is the first to capitalize on the ubiquity of smartphones to large-scale dissemination of CBT-based strategies aimed at preventing depression in non-clinical populations.

**Trial registration:**

ClinicalTrials.gov: NCT02783118. Registered on 26 May 2016.

**Electronic supplementary material:**

The online version of this article (doi:10.1186/s13063-016-1740-3) contains supplementary material, which is available to authorized users.

## Background

About 350 million people of all ages are affected by depression worldwide [1]. In 2008, the World Health Organization (WHO) estimated depression to be the third largest contributor to the global disease burden (GDB) globally and the number one contributor to disease burden in developed countries [2]. In 2010, depression was estimated to be the second largest contributor to GDB [3] and, by 2030, it is expected to become its leading contributor [4].

In the USA alone, depression costs $81 billion [5], while in Europe it reaches about €118 billion per year [6, 7].

Although effective treatments for this condition do exist [8, 9], they do not seem to help in decreasing the prevalence of depression or the associated GDB [10–12]. One of the most important factors contributing to this reality is the fact that fewer than half of those in need of treatment (and in many countries, fewer than 10%) receive adequate care [1]. Some reasons for this are the limited availability of adequately trained therapists, stigma, or logistical costs [13, 14]. Thus, increasing treatment accessibility for the affected people can have the potential to reduce the GDB associated with depression.

Besides increasing the treatment availability for people experiencing depression, it is also very important to *prevent* the development of depressive symptoms. Subthreshold depression has been shown to predict the onset of full-blown depressive episodes [15]. In addition, research shows that about 80% of people who suffer a first major depressive episode will have at least one more [16], with a lifetime average of four episodes [17]. Even when successfully treated, depressive symptoms are recurrent in about 40% of cases within the first 2 years after treatment [18]. If we consider that (1) although antidepressant medication is effective, only 50–70% of patients respond to it [19], and (2) response rates to cognitive behavioral therapy (CBT), one of the most empirically validated psychological treatments for depression, are also around 60% for moderate to severe depression [20], preventing depressive symptoms — along with striving for innovative strategies to increase the efficacy of the available treatments — becomes critical.

### Framework and rationale of the study

Depression prevention efforts represent a global priority [21]. A range of interventions, including educational, psychotherapeutic, pharmacological, and lifestyle and nutritional approaches, has been shown to have some utility in preventing the development of this condition [22, 23]. However, considering the global challenge raised by depression, there is a need to develop strategies that produce more enduring preventative effects [21]. Using insights from the theories at the basis of the best empirically supported psychological treatments available nowadays, such strategies should comprehensively target (1) *cognitive factors* such as negative attitudes or cognitive inflexibility [24–27], and (2) *social stressors* and *behavioral factors* that can activate and/or exacerbate depressive symptoms [21]. In addition, innovative methods designed to facilitate the efficient implementation and dissemination of evidence-based prevention strategies are needed. Cuijpers et al. [21] suggested that the incorporation of new media, such as e-mental health programs and smartphone solutions, in prevention strategies could prove invaluable in facilitating large-scale implementation of prevention strategies. A similar recommendation has also been advanced by the WHO, which, in its Mental Health Action Plan 2013–2020, advises ”the promotion of self-care, for instance, through the use of electronic and mobile health technologies” [28].

Recent advances in mobile technology and Internet use have resulted in a niche industry for mental health apps, especially for depression. Searching for “depression” in the Apple Store or Google Apps Store yields hundreds of hits (apps) related to this condition, covering aspects such as measuring depressive symptoms, tracking mood, helping people to think more positively or expressing their gratitude on a regular basis, monitoring dysfunctional thoughts and identifying distorted thinking patterns, challenging dysfunctional thoughts, promoting more diversified activity patterns, and so on. However, how useful they are in terms of preventing or ameliorating depressive symptoms has yet to be established. Preliminary findings showed promising results in reducing subthreshold or mild to moderate depression [29–34]. Still, such studies are small (pilot studies) and few in number, and have not been replicated [35].

The evidence supporting the use of such apps is building up, but additional consistent experimental work is needed before we can draw clear conclusions. To our knowledge, no formalized study has attempted to test the utility of smartphone apps in *preventing* depression. Targeting risk factors for depression, such as cognitive vulnerability [27, 36, 37] and subthreshold depressive symptomatology [15], could represent a first step in building well-informed preventative online programs for this condition (see also [38]).

### Objectives

The aim of this exploratory study is to test a newly developed app for decreasing cognitive vulnerability in the general adult population, and, respectively, reducing minimal symptoms of depression. To this end, two samples of participants will be recruited: (1) participants free of depressive symptoms, and (2) participants presenting with mild depression symptoms. Within each sample, the participants will be randomized to an active group (i.e., a group receiving preventive intervention) and to a wait-list control, respectively. The clinical utility of the application will be assessed by comparing randomized group scores within each sample type with respect to (1) depressive symptom level, (2) negative and positive effects, (3) satisfaction with life, and (4) behavioral and cognitive vulnerability factors involved in the onset of depression.

This study will capitalize on one of the main advantages of smartphones, namely broad accessibility. Simply, through this app, we aim to target the majority of smartphone owners, in an attempt to (1) remit minimal depressive symptoms (to prevent their escalation), (2) decrease general cognitive vulnerability for depression and promote protective behaviors, even in the absence of any symptoms, and (3) reduce general negative effect and increase satisfaction with life. This initiative is congruent with recent recommendations in the literature [39], as well as with basic CBT principles and empirical evidence, which indicate that supporting individuals in coping with non-clinical psychological distress can prevent symptoms from reaching clinical significance [40, 41]. In addition, this app is designed to promote continuous engagement, by incorporating tailoring, customization, and gamification elements, aimed at nurturing intrinsic motivation to use the app. Previous data show that one of the main problems with computerized CBT-based interventions is premature dropout [42–44]. Therefore, though effective and largely accessible [45, 46], these interventions often do not reach their maximum potential because users prematurely give up using them.

Borrowing from CBT principles, with a therapeutic package inspired from rational emotive behavior therapy (REBT) [47, 48], a new application (app) has been developed and will be tested to examine its benefits in decreasing cognitive vulnerability and reducing mild depressive symptomatology as risk factors for depression.

## Methods/design

### Trials design

These are two-arm, parallel-group, randomized controlled exploratory trials examining the effects of an mHealth intervention, developed to decrease cognitive vulnerability and reduce mild depressive symptomatology, compared to wait-list control in people with no symptoms (study 1) and people with mild symptoms (study 2). The participants in the mHealth condition will be followed up for 12 months, and those in the wait-list control group will receive the intervention after they have completed the post-intervention assessment at 4 weeks. The design of the trials is similar to and compliant with the Consolidated Standards of Reporting Trials (CONSORT) guidelines [49] and with the Standard Protocol Items: Recommendations for Interventional Trials (SPIRIT) statement 2013 [50] (see also Additional file 1: SPIRIT checklist and schedule of enrollment, interventions, and assessments).

### General description of the tested application

The app to be tested, available for download for iOS and Android, has the following features:

1. Profile and overview section: The first screen of the application (see Fig. 1) after the login summarizes the information about the user’s activity in a simple and attractive format. On this screen, the users can choose and personalize an avatar and check the current “Energy” level reached within the application (see details below).

**Fig. 1:**
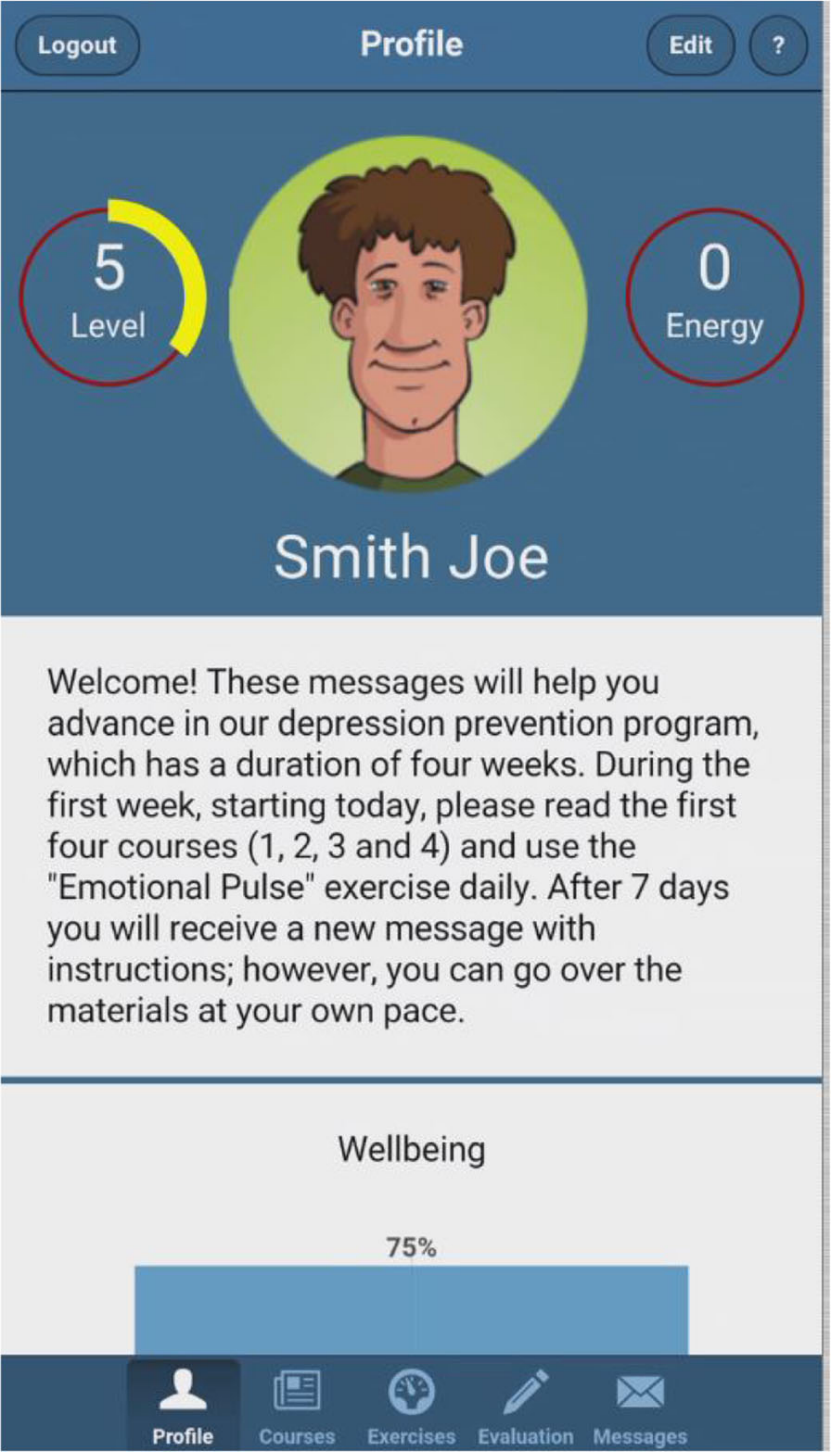
App screenshot showing an example of the profile and overview section
  As non-depressed users could be less motivated to use an app designed for clinical use than people suffering from depression, it is important to keep them interested. To this end, the app incorporates an attractive design in both the visual presentation and flow of activities [51, 52]. Borrowing from gamification strategies [53], the app integrates two graphical representations similar to role-play games: Energy and Level. ”Energy” represents a percentage value that decreases over time if the app is not used. The incentive is to keep the Energy level as close to 100% as possible. Reading articles, completing exercises, and filling out questionnaires are all rewarded with an increase in Energy. ”Level” represents the user’s progress through the educational articles — reading each article increases the Level by 1. The goal is to reach the maximum level (i.e., Level 12). This overview also includes a chart representing the user’s progress in improving his/her mood over time, measured via a scale in the evaluation section (described below).
2. Psychoeducation section: This section contains articles and self-help materials covering topics such as the causes of depression, the impact of dysfunctional thinking patterns and unproductive behaviors, healthy and unhealthy negative emotions, as well as a series of educational videos about relaxation techniques.
3. Exercises: The application contains a series of guided cognitive restructuring exercises, following the ABCDE model of emotional disturbance [48, 54]. These exercises are based on a model where each emotion is assigned to several activated events and the specific underlying rational/irrational beliefs — so that the user is automatically guided through rational thinking patterns, without the need for therapist feedback (see Fig. 2). This is an important aspect of prevention programs, as easy access and “non-consumable” services could be easily provided to a great number of users [38]. Completing each activity and exercise is rewarded graphically through a notification and an increase of the Energy percentage.

**Fig. 2:**
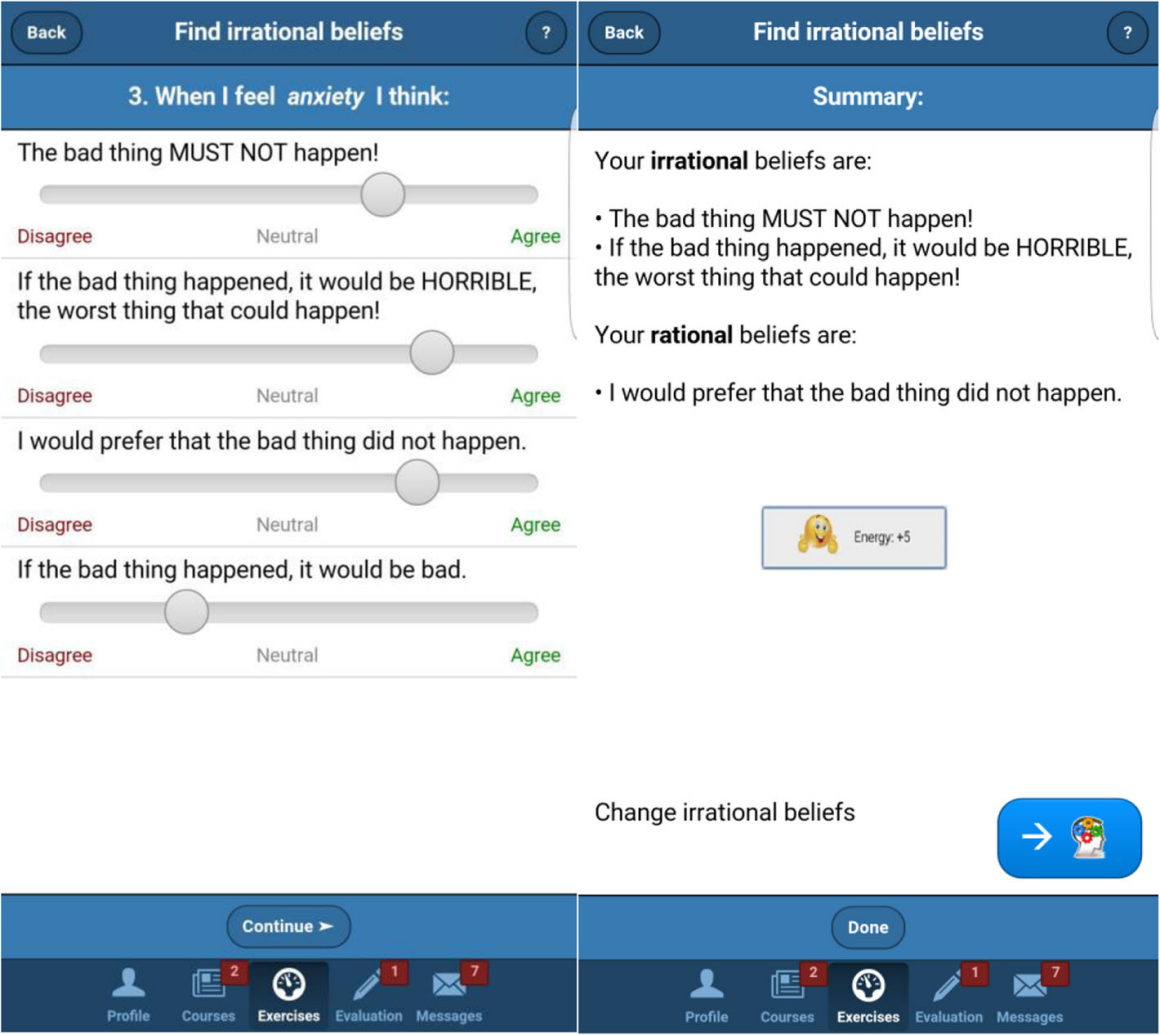
App screenshots showing the “Find irrational beliefs exercise”
4. Evaluation section: Without leaving the application, the user can fill out questionnaires related to the study being conducted, along with a mood inventory. The mood inventory updates the progress graph on the profile section, described earlier. Whenever a new evaluation is due, the user is notified and rewarded with more Energy after completing it.
5. Messages section: Each week the users are encouraged, via automated messages, to read articles and use the exercises available. They are also reminded to complete that week’s assessment. Moreover, this section allows them to send messages to the research team regarding technical difficulties or other obstacles encountered in using the application’s interface.

### Study setting

The study population consists of Romanian adults with (1) no depressive symptomatology (first sample) or (2) with mild depressive symptomatology (second sample). The study is being implemented through the Babeș-Bolyai University, Cluj-Napoca, Romania.

#### Eligibility criteria

##### Inclusion criteria for the first trial

Healthy Romanian-speaking adults (18 years or older) with access to a computer, a smartphone (Android or iOS), and the Internet are included in the study. The Structured Clinical Interview for DSM-IV (SCID) [55], specifically the Overview module, will be used to determine the general clinical status of the participants. A clinical psychologist will conduct a short interview aimed to screen for exclusion criteria (see below). Information on drug use and physical and psychological treatment status (past and present) will be collected. In addition, to be included in the study, participants should report a Patient Health Questionnaire (PHQ-9) score no greater than 4.

##### Exclusion criteria for the first trial

Participants undergoing therapy (i.e., medication and/or psychotherapy), presenting with substance abuse problems, psychotic symptoms, organic brain disorders (e.g., dementia), self-injury or harming others, or serious legal or health issues that would prevent them from using the app, as well as participants reporting scores greater than 1 to Question 9 (suicidal ideation) on the PHQ-9 [56] are excluded.

##### Inclusion and exclusion criteria for the second trial

The inclusion criteria for the second trial will be the same as those for the first trial, except for the PHQ-9 cut-off. More specifically, to be included in the trial, participants should obtain a PHQ-9 score above 4, but no larger than 9. Exclusion criteria for the second trial are identical to those for the first trial.

### Study conditions: *the mHealth intervention*

The app being tested has two main components: courses and exercises. (1) *Courses* represent the psychoeducational and therapeutic background of the program and include information on the following topics: depression, psychological vulnerability, CBT, healthy and unhealthy negative emotions, rational and irrational thoughts, how behaviors contribute to depression, sleep hygiene, social support, and relaxation techniques. (2) *Exercises* use the information presented in the courses and follow the structure of a regular therapy session and/or therapeutic homework.

The following exercises are included in the app:

1. The *Emotional Pulse* exercise registers the user’s current activity and emotions (healthy or unhealthy).
2. The *Sticky Notes* exercise consists of a series of tasks targeting behavioral activation (e.g., setting goals, building a list of rewards/pleasant activities).
3. The *Find Irrational Thoughts* exercise helps to identify thoughts behind the emotions found with the Emotional Pulse exercise.
4. The *Change Irrational Thoughts* exercise challenges negative/irrational thinking behind unhealthy emotions and replaces it with healthy, flexible, and functional thoughts.

The app can be used standalone, i.e., independently by healthy or mildly depressed participants, without therapist guidance.

### The wait-list control group

Participants in the delayed intervention group are placed on a wait list for 4 weeks; then they are given full access to the application.

### Outcomes and measures

#### Primary outcomes

For the first trial, involving a healthy sample of participants, cognitive vulnerability factors as defined within the CBT paradigm (i.e., dysfunctional cognitions, irrational beliefs, and negative automatic thoughts) [24–26] constitute the primary outcomes. For the second trial, involving a sample of mildly depressed participants, the level of depressive symptomatology constitutes the primary outcome.

#### Secondary outcomes

For the first trial, the level of depressive symptomatology, the general positive and negative effects, and satisfaction with life constitute the secondary outcomes. Besides depressive symptomatology, emotionality and quality of life outcomes have been included to assess the efficacy of the application on a broader spectrum of psychological variables. For the second trial, the general positive and negative effects and satisfaction with life constitute the secondary outcomes.

#### Other outcomes

For the first trial, behavioral activation and/or avoidance as possible predictors of the outcomes will be examined. For the second trial, dysfunctional cognitions, irrational beliefs, and negative automatic thoughts (conceptualized as mechanisms of change) and behavioral activation and/or avoidance will be considered as possible predictors of the outcomes.

Satisfaction with the application and data regarding the app usage are also assessed in both trials.

The instruments used for each of the above-mentioned outcomes are presented below.

#### Screening measures

The Overview module of the SCID [55] will be used to determine the general clinical status of the participants. Information on drug use and physical and psychological treatment status (past and present) will be collected. The SCID has been adapted for use on the Romanian population [57].

The PHQ-9 [56] is a nine-question instrument designed to correspond to the *Diagnostic and Statistical Manual of Mental Disorder,* 4th edition, Revised Text (DSM-IV-TR) [58] diagnostic criteria for major depressive disorder. Respondents rate the items from 0 to 3 according to the frequency of their experience over the previous 2-week period (0: not at all; 3: nearly every day). The score can then be interpreted as indicating the depression severity (no depression, mild, moderate, moderately severe, or severe depression). The PHQ-9 has been adapted into Romanian for the purposes of the current study.

#### Measures of primary, secondary, and other outcomes

The level of depressive symptomatology is assessed with the Center for Epidemiologic Studies Depression Scale-Revised (CESD-R) [59, 60]. The CESD-R is a 20-item self-report instrument which measures symptoms of depression in nine different categories: sadness (dysphoria), loss of interest (anhedonia), appetite, sleep, thinking/concentration, guilt (worthlessness), tiredness (fatigue), movement (agitation), and suicidal ideation. Participants rate each item on a five-point Likert scale, from 0 (not at all or less than one day) to 4 (nearly every day for 2 weeks) to indicate how they felt or behaved during the last week or so. The Total CESD-R Score is calculated as a sum of responses to all 20 questions. The CESD-R exhibited good psychometric properties, including high internal consistency, strong factor loadings, and theoretically consistent convergent and divergent validity with anxiety, schizotypy, and positive and negative effects [61]. The CESD-R has been adapted to Romanian for the purposes of the current study.

*The Positive and Negative Affect Scale* (PANAS) [62] is a 20-item self-report questionnaire designed to assess mood. It consists of 10 items that address positive affect (PA) and 10 items that address negative affect (NA). Participants rate each item on a five-point Likert scale, from 1 (very slightly/not at all) to 5 (extremely) to indicate how they felt during the indicated timeframe. The PANAS can be used to assess mood on various time scales by altering the instructions. For the purposes of this study, we have used a 2-week timeframe. The validity and internal consistency of the PANAS are good, with test–retest reliability being the highest for the “general” temporal instruction. The PANAS has been used previously on the Romanian population and was found to have adequate psychometric properties [63, 64].

*Satisfaction with Life* (SWL) [65] is a five-item scale designed to measure global cognitive judgements of one’s life satisfaction. Participants rate each of the five items using a seven-point scale that ranges from 7 (strongly agree) to 1 (strongly disagree). The SWL has been shown to be a valid and reliable measure of life satisfaction which can be used with a wide range of age groups [66, 67]. The SWL scale has been adapted to Romanian for the purposes of the current study.

*The Dysfunctional Attitude Scale* (DAS) [68] was designed to measure the intensity of dysfunctional attitudes that, according to the cognitive theory of depression [25], contribute to vulnerability for depression. For the purpose of our study, we used the short form of this scale. The Dysfunctional Attitude Scale - Short Form (DAS-SF) [69] consists of two subscales: “dependency” (6 items) and “perfectionism/performance evaluation” (11 items). The 17 items are rated on a seven-point Likert scale, from 1 (total disagreement) to 7 (total agreement). The DAS-SF possesses good psychometric properties in terms of model fit, reliability, and convergent construct validity [69]. The DAS-SF has been adapted to Romanian for the purposes of this study.

*The Beliefs Scale* (BS) [70] measures irrational beliefs. It consists of 20 items, and responders indicate how much they agree or disagree with each item using a five-point Likert scale that ranges from 1 (strongly disagree) to 5 (strongly agree). The BS shows good psychometric properties regarding construct and discriminant validity [71]. This scale has been translated into Romanian for the purposes of this study.

*The Automatic Thoughts Questionnaire* (ATQ) [72] is a 15-item self-report measure used to assess depression-related cognitions. Participants rate, on a five-point Likert scale from 1 (never) to 5 (almost all the time), how frequently they have had a given thought over the past week. A higher score shows a higher frequency of automatic thoughts. The psychometric properties of the ATQ have been adequately demonstrated in previous studies [73]. The ATQ has been successfully used previously on the Romanian population [74–76].

*The Behavioral Activation for Depression Scale - Short Form* (BADS-SF) [77] is an instrument designed to be administered weekly to measure changes in avoidance and activation over the course of the Behavioral Activation (BA) treatment for depression. The BADS consists of nine items grouped into two subscales (Activation and Avoidance/Rumination). Respondents rate each item on a seven-point Likert scale ranging from 0 (not at all) to 6 (completely). The scale has good psychometric properties [78]. The BADS-SF has been translated into Romanian for the purposes of this study.

*Satisfaction with the Application Scale* was specifically designed for this study. It consists of 10 items that assess users’ satisfaction with the application, its difficulty level, attractiveness, and subjective utility. The first 8 items are rated on a three-point scale, ranging from 0 to 2. Each response scale is personalized to the content of the item (e.g., How attractive did you find the exercises included in the application? - 0 = rather unattractive, 1 = attractive enough, 2 = very attractive). Item 9 assesses the application globally, with the participant being asked to give an overall grade between 1 (minimum) and 10 (maximum). Item 10 asks the participants if they would recommend the application to a friend ("yes" or "no").

*The Application Use Scale* was also developed specifically for this study. It consists of 8 items that assess weekly quantitative app usage aspects: the effort invested in homework (1 item), number of practiced exercises (1 item), number of read courses (1 item), frequency of general application use (1 item), and frequency of every exercise use (4 items).

Two Romanian English-proficient post-doctoral clinical psychologists, with good knowledge of the constructs measured, independently translated all the instruments adapted into Romanian for the purposes of the present study. Disagreements were resolved through discussions between the translators. A senior clinical psychologist and the principal investigator also reviewed and approved the final versions.

#### Participant timeline

Possible participants are invited to access the study's website [79] and, after carefully reading the information package, they are instructed to create an online account. The participants are then asked to answer a few demographic questions (i.e., report on their age, education, status on the labor market, and marital/co-habitation status) and complete the PHQ-9 to determine their eligibility for further evaluation. If eligible, a screening procedure is implemented. A short telephone interview screens out those people whose interest in the study is motivated by issues other than their mood (e.g., curiosity, practical/life problems, need for a psychological assessment). Applicants who do not meet the inclusion criteria are informed via email. They are thanked for their interest, given a summary score and interpretation for their PHQ-9 score, and encouraged to discuss their problems with a professional, if necessary. Information on how to reach a clinical psychologist or psychotherapist is also provided.

**Fig. 3:**
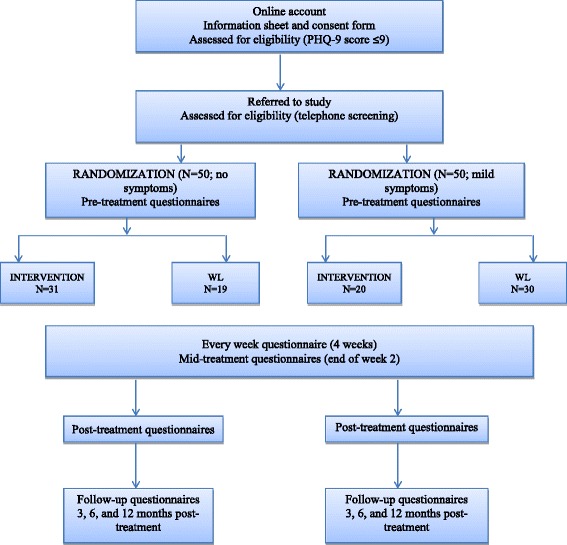
CONSORT flow diagram [49] showing subject allocation to the study conditions

**Fig. 4:**
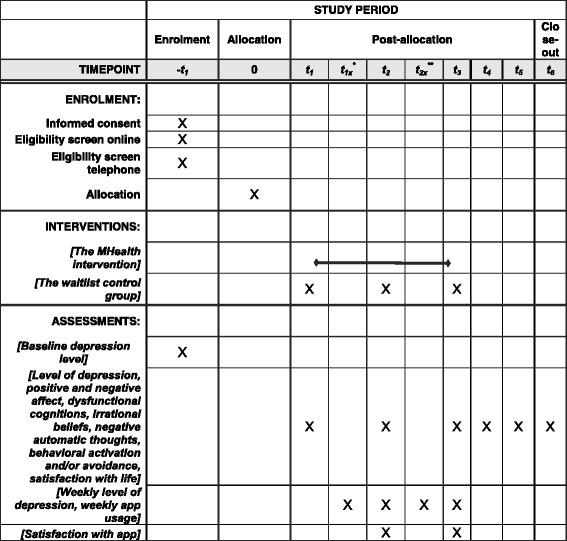
Schedule of enrollment, interventions, and assessments. *t_1x_ and t_2x_ represent weekly separate assessments

Participants meeting inclusion criteria will be randomly assigned to one of the two conditions: immediate online intervention condition (mHealth intervention) or the delayed-intervention condition (see the flow diagram in Fig. 3). Subsequent assessments consist of all the above instruments. Figure 4 shows the schedule of enrollment, interventions, and assessments.

The participants in the mHealth intervention are assessed at pre-intervention (time 1, baseline), at mid-intervention (time 2, 2 weeks after baseline), at post-intervention (time 3, 4 weeks after baseline), and at 3, 6, and 12 months post-intervention (times 4, 5, and 6). The participants in the delayed-intervention condition are assessed before the waiting period (time 1, baseline), at mid-waiting period (time 2, 2 weeks after baseline), and at post-waiting period (time 3, 4 weeks after baseline).

Participants assigned to the mHealth intervention will be given access to the online application along with explicit instructions regarding the use of all of its sections. Participants will be given 4 weeks to complete the intervention, during which time weekly messages will be sent out to them. Messages include regular assignments designed for a complete and thorough use of the application's courses and exercises, and encourage the review of materials whenever possible. However, participants are free to use the application at their own pace.

After each week, participants’ application use is evaluated (see the *Application Use Scale* described above). Similarly, after each week of using the application, the participants are required to complete the CESD-R for close monitoring of their depressive symptomatology and individualized feedback on their emotional state.

Participants in the delayed intervention group are placed on a wait list for 4 weeks.

### Sample size

The sample size was estimated considering the exploratory nature of the two trials, in terms of investigating the feasibility of the design and methodology employed, as well as app-related usability aspects. As such, we followed current research practice exploratory protocols. For example, in an audit of sample sizes for pilot and feasibility trials being undertaken in the UK, Billingham, Whitehead, and Julious [80] have found that the median pilot study sample size for two-arm trials was 30 participants per arm, for continuous endpoints. Taking this into consideration, as well as promising results (i.e., large within-group effect sizes) reported by previous studies testing mobile phone applications for depression [81], a total number of 50 participants per study (i.e., 50 participants without depressive symptomatology and 50 participants with mild depressive symptomatology) was anticipated.

### Recruitment

Possible participants are approached through presentations at various events and ads in the media. Additionally, clinicians from the private practice area are contacted for referrals. Those interested in using the application are asked to provide their contact details and are subsequently contacted via email, at which point the enrollment procedure is described.

### Assignment to study group

The participants are assigned to one of the two trials, depending on their depressive symptomatology level (i.e., PHQ-9 score). Using the software Randomizer.org, participants are then randomly distributed to one of the trials’ conditions. Randomization is performed by a research assistant using a simple (unrestricted) randomization sequence that assigns two unique numbers per participant; the number assigned is either 1 or 2, according to the number of experimental conditions. To conceal the allocation mechanism, the same research assistant will monitor the assessments and allow access to the application for the participants in the wait-list control group, after 1 month. The principal investigator and the statisticians running the data analysis will remain blinded to the study condition until the completion of the study.

### Data collection, management, and analyses

#### Data collection methods

The data are collected within the application, in the Evaluation section, and transferred to our servers using an encrypted, secure (https) industry-standard transfer protocol.

The following strategies will be implemented to promote participant retention and follow-up completion. First of all, participants are reminded every week of the specific assessment that needs to be completed, through the Messages section, and are rewarded with more ”Energy” after completing it. Participants are also told that the weekly completion of the mood questionnaires allows the app to create a personalized profile of their mood (through the profile section) and, thus, give individualized feedback on their emotional state. Furthermore, the messages that the participants receive every week comprise gradual steps in covering the app’s content and exercises — every message refers to a new content and/or new exercise to be covered during that week, explaining its purpose and possible use, and, at the same time, encouraging the participant to come back the next week. Lastly, for the follow-up assessments, participants will receive new messages reminding them that the free use of the app was possible because of a research grant, for which the collection of this data is essential.

#### Statistical methods

Improvement in the primary outcomes measures scores within and between the groups (for each study) will be examined using mixed-effects linear regression analysis with a random intercept and slope over time (three assessments: baseline, mid-intervention, post-intervention) and fixed effects for intervention assignment. The 3-, 6-, and 12-month follow-up data will be analyzed in a separate fixed-effects model. The main analysis will compare groups (experimental and wait-list) in terms of cognitive vulnerability factors for the first trial, or level of depressive symptoms for the second trial. When analysis of secondary/other outcomes is performed, the error probability will be adjusted according to the number of group comparisons performed.

An intent-to-treat analysis will also be performed to examine participants who dropped out prematurely.

App usage data and user activity will be examined in an exploratory manner by comparing the participants’ self-reported activity with their actual app usage as captured by our software. This wealth of data will allow the possibility of pinpointing any obstacles to user adherence to the protocol, and will shed light on the reasons for dropouts or irregular usage patterns.

### Monitoring study implementation

Two clinical psychologists, members of the study team, screen for the risk of unintended effects or harm to the participants (i.e., clinically significant increase in depressive symptomatology, as measured by the CESD-R). The psychologists monitor the weekly online evaluations and clinically interpret the CESD-R score of every participant. If the participant does not complete the CESD-R evaluation, she/he is contacted by telephone. If necessary, the supervisor can decide to interrupt the participant's access to the application and make a further referral.

### Ethics and dissemination

The ethics commission at Babeș-Bolyai University has approved this study. Although it does not involve the delivery of a treatment to depressed people, it does target individuals with mild depressive symptomatology who may be at risk for developing depression. Hence, there are ethical concerns that need to be addressed. Firstly, the initial screening process and the exclusionary criteria single out people at risk for suicide, who are immediately referred to the appropriate clinical care. Secondly, if a participant’s condition worsens during the use of the application, the clinical team can opt to interrupt his/her access to the application and make a further referral or recommendation. Regarding confidentiality, special attention has been given to securing the online data collection and storage. Sensitive clinical data are collected either through a web browser (during the signup procedure) or from the mobile application. Both these means communicate the data to the server over industry-standard secure connections (i.e., https). The server stores the data in a database that is not publicly accessible. The system running our applications is kept up to date to prevent intrusions. Access to the electronic data is password-protected, and the passwords are changed regularly. The clinical screening reports contain no personal identifying information.

### Dissemination policy

Preliminary features of the application and its intended purpose have been presented at several conferences, nationally and internationally. Also, this research is being promoted and constantly updated in terms of progress and dissemination through its designated website [82]. The two trials’ results will be submitted for publication in peer-reviewed journals, focusing on (1) feasibility and usability results and (2) primary and secondary outcome results. New presentations at international conferences on the topic of e-health solutions are also being considered.

## Discussion

This exploratory study describes two randomized clinical trials testing a smartphone app aimed at reducing cognitive vulnerability and mild depressive symptoms, as risk factors for the onset of depression. The two trials’ targets are (1) the general population and (2) a population with minimal depressive symptoms. This research effort is motivated by the global challenge that depression raises. Depression prevalence and costs have continuously increased over the years [1, 2, 4, 83]. Subthreshold depression also has a high prevalence [84, 85], is associated with considerable individual and societal costs [86, 87], and predicts future full-blown depressive episodes [15, 88]. Depressive symptoms usually do not remit without treatment [89], yet access to interventions is limited [1], responses to available treatments are suboptimal [19, 20], and recurrence rates are substantial [18]. Therefore, large-scale preventative strategies *before* people experience depressive disorders (when they are in need for full treatment) are needed.

To our knowledge, this is the first attempt to capitalize on the ubiquity of smartphones to large-scale dissemination of CBT-based strategies aimed at preventing depression in non-clinical populations. By generating the first indirect evidence related to the potential value of apps for preventing depression, through decrease in cognitive vulnerability and reduction of depressive symptoms, this study can add substantially to the relatively small body of evidence indicating that smartphone apps could be successfully used to manage depression [35, 39, 90]. The app developed for this study is designed to decrease general cognitive vulnerability and promote engagement in protective, adaptive activities, while counteracting the tendency of premature dropout (through gamification and customization). This study capitalizes on targeting cognitive vulnerability factors as mechanisms of change. Notably, studies conducted until now have focused almost exclusively on testing the apps clinical efficacy in decreasing symptoms. The investigation of the mechanisms of change in relation to technologically mediated CBT preventive/intervention strategies seems to be grossly under-investigated. By including two types of samples recruited from a general, non-clinical population (i.e., symptom-free participants, and participants showing minimal symptoms of depression, respectively), this research may be able to add support to the assumption that symptoms remission/lowering is explained by a decrease in cognitive vulnerability [25]. Although the study design does not allow the provision of irrefutable support for such an assumption, the results do have the potential of guiding future research.

There are, however, limitations of this study. The main one is related to the control group. Ideally, an active placebo control should be included. Yet, because there are no studies investigating the potential utility of apps in preventing depressive symptoms in a non-clinical population, a simple wait-list control was included. Nevertheless, this implies that, if the expected results are obtained, the possibility that they are explained by the simple usage of the app, rather than the app content, will not be ruled out. However, because a decrease in symptoms is not the main or the sole indicator of the app’s efficacy but, rather, may be a possible change in cognitive vulnerability factors in a population free of marked psychological distress, the authors believe that the simple usage of the app is unlikely to explain eventual positive results. However, future studies should strive to contrast the “active” app with a “sham” app (i.e., an alternative app which looks similar, but does not target cognitive vulnerability factors associated with depression) in order to derive firm conclusions.

## Trial status

Participant recruitment began on 7 June 2016. Randomization of the participants was performed on 15 June 2016.

## Additional file

Additional file 1: SPIRIT 2013 checklist: recommended items to address in a clinical trial protocol and related documents. (DOC 122 kb)

## Abbreviations

ATQ: Automatic Thoughts Questionnaire
BADS-SF: Behavioral Activation for Depression Scale - Short Form
BS: Beliefs Scale
CBT: Cognitive behavioral therapy
CESD-R: Center for Epidemiologic Studies Depression Scale-Revised
DAS: Dysfunctional Attitude Scale
DAS-SF: Dysfunctional Attitudes Scale - Short Form
DSM-IV-TR: Diagnostic and Statistical Manual of Mental Disorders 4th edition
GDB: Global disease burden
PANAS: Positive and Negative Affect Scale
PHQ-9: Patient Health Questionnaire, 9-item version
REBT: Rational emotive behavior therapy
SWL: Satisfaction with Life
WHO: World Health Organization

## References

[1] World Health Organization. Depression. 2016. http://www.who.int/mediacentre/factsheets/fs369/en/. Accessed 8 Apr 2016.

[2] World Health Organization. Policies and practices for mental health in Europe. Meeting the challenges. 2016. http://www.euro.who.int/en/publications/abstracts/policies-and-practices-for-mental-health-in-europe.-meeting-the-challenges. Accessed 8 Apr 2016.

[3] Ferrari AJ, Charlson FJ, Norman RE, Patten SB, Freedman G, Murray CJL, Vos T, Whiteford HA (2013). Burden of depressive disorders by country, sex, age, and year: findings from the Global Burden of Disease study 2010. PLoS Med.

[4] World Health Organization. Global burden of mental disorders and the need for a comprehensive, coordinated response from health and social sectors at the country level. 2011. http://apps.who.int/gb/ebwha/pdf_files/EB130/B130_9-en.pdf. Accessed 14 Apr 2016.

[5] Greenberg PE, Kessler RC, Birnbaum HG, Leong SA, Lowe SW, Berglund PA, Corey-Lisle PK (2003). The economic burden of depression in the United States: how did it change between 1990 and 2000?. J Clin Psychiatry.

[6] Olesen J, Gustavsson A, Svensson M, Wittchen H-U, Jönsson B (2012). The economic cost of brain disorders in Europe. Eur J Neurol.

[7] Sobocki P, Jönsson B, Angst J, Rehnberg C (2006). Cost of depression in Europe. J Ment Health Policy Econ.

[8] Cuijpers P, Sijbrandij M, Koole SL, Andersson G, Beekman AT, Reynolds CF (2013). The efficacy of psychotherapy and pharmacotherapy in treating depressive and anxiety disorders: a meta-analysis of direct comparisons. World Psychiatry.

[9] Hofmann SG, Asnaani A, Vonk IJJ, Sawyer AT, Fang A (2012). The efficacy of cognitive behavioral therapy: a review of meta-analyses. Cogn Ther Res.

[10] Greenberg PE, Fournier A-A, Sisitsky T, Pike CT, Kessler RC (2015). The economic burden of adults with major depressive disorder in the United States (2005 and 2010). J Clin Psychiatry.

[11] Hidaka BH (2012). Depression as a disease of modernity: explanations for increasing prevalence. J Affect Disord.

[12] Lépine J-P, Briley M (2011). The increasing burden of depression. Neuropsychiatr Dis Treat.

[13] Cuijpers P (2011). The patient perspective in research on major depression. BMC Psychiatry.

[14] Goldman LS, Nielsen NH, Champion HC (1999). Awareness, diagnosis, and treatment of depression. J Gen Intern Med.

[15] Cuijpers P, Smit F (2004). Subthreshold depression as a risk indicator for major depressive disorder: a systematic review of prospective studies. Acta Psychiatr Scand.

[16] Fava GA, Park SK, Sonino N (2006). Treatment of recurrent depression. Expert Rev Neurother.

[17] Limosin F, Mekaoui L, Hautecouverture S (2007). Stratégies thérapeutiques prophylactiques dans la dépression unipolaire. Presse Med.

[18] Boland R, Keller M, Gotlib IH, Hammen HL (2009). Course and outcome of depression. Handbook of Depression.

[19] Gartlehner G, Hansen RA, Thieda P, DeVeaugh-Geiss AM, Gaynes BN, Krebs EE, Lux LJ, Morgan LC, Shumate JA, Monroe LG, Lohr KN (2007). Comparative effectiveness of second-generation antidepressants in the pharmacologic treatment of adult depression.

[20] DeRubeis RJ, Hollon SD, Amsterdam JD, Shelton RC, Young PR, Salomon RM, O’Reardon JP, Lovett ML, Gladis MM, Brown LL, Gallop R (2005). Cognitive therapy vs medications in the treatment of moderate to severe depression. Arch Gen Psychiatry.

[21] Cuijpers P, Beekman ATF, Reynolds CF (2012). Preventing depression. JAMA.

[22] Muñoz RF, Beardslee WR, Leykin Y (2012). Major depression can be prevented. Am Psychol.

[23] Buntrock C, Ebert DD, Lehr D, Smit F, Riper H, Berking M, Cuijpers P (2016). Effect of a web-based guided self-help intervention for prevention of major depression in adults with subthreshold depression: a randomized clinical trial. JAMA.

[24] Beck AT (1963). Thinking and depression: I. idiosyncratic content and cognitive distortions. Arch Gen Psychiatry.

[25] Beck AT (1976). Cognitive therapy and the emotional disorders.

[26] Ellis A (1957). Rational psychotherapy and individual psychology. J Individ Psychol.

[27] Haeffel GJ, Rozek DC, Hames JL, Technow J (2011). Too much of a good thing: testing the efficacy of a cognitive bias modification task for cognitively vulnerable individuals. Cogn Ther Res.

[28] World Health Organization. Mental Health Action Plan: 2013–2020. http://apps.who.int/iris/bitstream/10665/89966/1/9789241506021_eng.pdf. Accessed 11 Apr 2016.

[29] Birney AJ, Gunn R, Russell JK, Ary DV (2016). MoodHacker mobile Web app with email for adults to self-manage mild-to-moderate depression: randomized controlled trial. JMIR Mhealth Uhealth.

[30] Burns MN, Begale M, Duffecy J, Gergle D, Karr CJ, Giangrande E, Mohr DC (2011). Harnessing context sensing to develop a mobile intervention for depression. J Med Internet Res.

[31] Kauer SD, Reid SC, Crooke AHD, Khor A, Hearps SJC, Jorm AF, Sanci L, Patton G (2012). Self-monitoring using mobile phones in the early stages of adolescent depression: Randomized controlled trial. J Med Internet Res.

[32] Proudfoot J, Clarke J, Birch M-R, Whitton AE, Parker G, Manicavasagar V, Harrison V, Christensen H, Hadzi-Pavlovic D (2013). Impact of a mobile phone and web program on symptom and functional outcomes for people with mild-to-moderate depression, anxiety and stress: a randomised controlled trial. BMC Psychiatry.

[33] Reid SC, Kauer SD, Khor AS, Hearps SJC, Sanci LA, Kennedy AD, Patton GC (2012). Using a mobile phone application in youth mental health — an evaluation study. Aust Fam Physician.

[34] Watts S, Mackenzie A, Thomas C, Griskaitis A, Mewton L, Williams A, Andrews G (2013). CBT for depression: a pilot RCT comparing mobile phone vs. computer. BMC Psychiatry.

[35] Anthes E (2016). Mental health: there’s an app for that. Nature.

[36] Haeffel GJ, Grigorenko EL (2007). Cognitive vulnerability to depression: exploring risk and resilience. Child Adolesc Psychiatr Clin N Am.

[37] Alloy LB, Abramson LY, Whitehouse WG, Hogan ME, Panzarella C, Rose DT (2006). Prospective incidence of first onsets and recurrences of depression in individuals at high and low cognitive risk for depression. J Abnorm Psychol.

[38] Muñoz RF, Cuijpers P, Smit F, Barrera AZ, Leykin Y (2010). Prevention of major depression. Annu Rev Clin Psychol.

[39] Bakker D, Kazantzis N, Rickwood D, Rickard N (2016). Mental health smartphone apps: review and evidence-based recommendations for future developments. JMIR Ment Health.

[40] Dobson D, Dobson KS (2009). Evidence-based practice of cognitive-behavioral therapy.

[41] Rohde P, Stice E, Shaw H, Brière FN (2014). Indicated cognitive behavioral group depression prevention compared to bibliotherapy and brochure control: acute effects of an effectiveness trial with adolescents. J Consult Clin Psychol.

[42] Christensen H, Griffiths KM, Farrer L (2009). Adherence in Internet interventions for anxiety and depression. J Med Internet Res.

[43] Kaltenthaler E, Parry G, Beverley C, Ferriter M (2008). Computerised cognitive-behavioural therapy for depression: systematic review. Br J Psychiatry J Ment Sci.

[44] So M, Yamaguchi S, Hashimoto S, Sado M, Furukawa TA, McCrone P (2013). Is computerised CBT really helpful for adult depression? A meta-analytic re-evaluation of CCBT for adult depression in terms of clinical implementation and methodological validity. BMC Psychiatry.

[45] Andrews G, Cuijpers P, Craske MG, McEvoy P, Titov N (2010). Computer therapy for the anxiety and depressive disorders is effective, acceptable and practical health care: a meta-analysis. PLoS ONE.

[46] Richardson T, Stallard P, Velleman S (2010). Computerised cognitive behavioural therapy for the prevention and treatment of depression and anxiety in children and adolescents: a systematic review. Clin Child Fam Psychol Rev.

[47] Dryden W, DiGiuseppe R (1990). A primer on rational-emotive therapy.

[48] Ellis A, Dryden W, DiGiuseppe R (2007). The practice of rational emotive behavior therapy.

[49] Schulz KF, Altman DG, Moher D, for the CONSORT Group (2010). CONSORT 2010 Statement: updated guidelines for reporting parallel group randomised trials. BMJ.

[50] Chan A-W, Tetzlaff JM, Altman DG, Laupacis A, Gøtzsche PC, Krleža-Jerić K, Hróbjartsson A, Mann H, Dickersin K, Berlin JA, Doré CJ, Parulekar WR, Summerskill WSM, Groves T, Schulz KF, Sox HC, Rockhold FW, Rennie D, Moher D (2013). SPIRIT 2013 statement: defining standard protocol items for clinical trials. Ann Intern Med.

[51] Crutzen R, De Nooijer J, Brouwer W, Oenema A, Brug J, De Vries NK (2009). A conceptual framework for understanding and improving adolescents’ exposure to Internet-delivered interventions. Health Promot Int.

[52] Ritterband LM, Thorndike FP, Cox DJ, Kovatchev BP, Gonder-Frederick LA (2009). A behavior change model for Internet interventions. Ann Behav Med.

[53] Zichermann G, Cunningham C (2011). Gamification by design: implementing game mechanics in web and mobile apps.

[54] Ellis A (1994). Reason and emotion in psychotherapy.

[55] First MB, Spitzer RL, Williams JBW, Gibbon M (1997). Structured Clinical Interview for DSM-IV (SCID).

[56] Kroenke K, Spitzer RL, Williams JB (2001). The PHQ-9: Validity of a brief depression severity measure. J Gen Intern Med.

[57] First MB, Spitzer RB, Gibbon M, Williams JBW (2007). Interviu Clinic Structurat Pentru Tulburarile Clinice de Pe Axa 1 a DSM — Versiune Clinica.

[58] American Psychiatric Association (2000). Diagnostic and statistical manual of mental disorders.

[59] Eaton W, Muntaner C, Smith C, Tien A, Ybarra M, Maruish M (2004). Center for Epidemiologic Studies Depression Scale: Review and revision (CESD and CESD-R). The use of psychological testing for treatment planning and outcomes assessment.

[60] Radloff LS (1977). The CES-D Scale: a self-report depression scale for research in the general population. Appl Psychol Meas.

[61] Van Dam NT, Earleywine M (2011). Validation of the Center for Epidemiologic Studies Depression Scale—Revised (CESD-R): pragmatic depression assessment in the general population. Psychiatry Res.

[62] Watson D, Clark LA, Tellegen A (1988). Development and validation of brief measures of positive and negative affect: the PANAS scales. J Pers Soc Psychol.

[63] Cristea IA, Matu S, Szentagotai Tătar A, David D (2013). The other side of rumination: reflective pondering as a strategy for regulating emotions in social situations. Anxiety Stress Coping.

[64] Levine EL, Xu X, Yang L-Q, Ispas D, Pitariu HD, Bian R, Ding D, Capotescu R, Che H, Musat S (2011). Cross-national explorations of the impact of affect at work using the State-Trait Emotion Measure: a coordinated series of studies in the United States, China, and Romania. Hum Perform.

[65] Diener E, Emmons RA, Larsen RJ, Griffin S (1985). The Satisfaction With Life Scale. J Pers Assess.

[66] Pavot W, Diener E, Colvin CR, Sandvik E (1991). Further validation of the Satisfaction with Life Scale: evidence for the cross-method convergence of well-being measures. J Pers Assess.

[67] Pavot W, Diener E (1993). Review of the Satisfaction With Life Scale. Psychol Assess.

[68] Weissman AN, Beck AT: Development and validation of the Dysfunctional Attitude Scale: a preliminary investigation. Paper presented at the 62^nd^ Annual Meeting of the American Educational Research Association, Toronto, Ontario, Canada, March 27–31, 1978.

[69] de Graaf LE, Roelofs J, Huibers MJH (2009). Measuring dysfunctional attitudes in the general population: The Dysfunctional Attitude Scale (form A) Revised. Cogn Ther Res.

[70] Malouff JM, Schutte NS (1986). Development and validation of a measure of irrational belief. J Consult Clin Psychol.

[71] Malouff JM, Valdenegro J, Schutte NS (1987). Further validation of a measure of irrational belief. J Ration Emotive Ther.

[72] Hollon SD, Kendall PC (1980). Cognitive self-statements in depression: development of an automatic thoughts questionnaire. Cogn Ther Res.

[73] Harrell TH, Ryon NB (1983). Cognitive-behavioral assessment of depression: clinical validation of the automatic thoughts questionnaire. J Consult Clin Psychol.

[74] David D (2007). Scale de Evaluare Clinică.

[75] Moldovan R, Cobeanu O, David D (2013). Cognitive bibliotherapy for mild depressive symptomatology: randomized clinical trial of efficacy and mechanisms of change. Clin Psychol Psychother.

[76] Vîslă A, Cristea IA, Szentágotai Tătar A, David D (2013). Core beliefs, automatic thoughts and response expectancies in predicting public speaking anxiety. Personal Individ Differ.

[77] Kanter JW, Mulick PS, Busch AM, Berlin KS, Martell CR (2007). The Behavioral Activation for Depression Scale (BADS): psychometric properties and factor structure. J Psychopatol Behav Assess.

[78] Manos RC, Kanter JW, Luo W (2011). The Behavioral Activation for Depression Scale-Short Form: development and validation. Behav Ther.

[79] BineDispus Study - official webpage. http://binedispus.ro.

[80] Billingham SA, Whitehead AL, Julious SA (2013). An audit of sample sizes for pilot and feasibility trials being undertaken in the United Kingdom registered in the United Kingdom Clinical Research Network database. BMC Med Res Methodol.

[81] Ly KH, Topooco N, Cederlund H, Wallin A, Bergström J, Molander O, Carlbring P, Andersson G (2015). Smartphone-supported versus full behavioural activation for depression: a randomised controlled trial. PLoS One.

[82] Project Dcombat: A Computerized Preventative and Therapeutic Intervention for Depression. http://dcombat.net/en/.

[83] CDC. Burden of mental illness - mental illness - mental health basics - mental health. 2013. http://www.cdc.gov/mentalhealth/basics/burden.htm. Accessed 14 Apr 2016.

[84] Kessler RC, Zhao S, Blazer DG, Swartz M (1997). Prevalence, correlates, and course of minor depression and major depression in the national comorbidity survey. J Affect Disord.

[85] Preisig M, Merikangas KR, Angst J (2001). Clinical significance and comorbidity of subthreshold depression and anxiety in the community. Acta Psychiatr Scand.

[86] Rapaport MH, Judd LL, Schettler PJ, Yonkers KA, Thase ME, Kupfer DJ, Frank E, Plewes JM, Tollefson GD, Rush AJ (2002). A descriptive analysis of minor depression. Am J Psychiatry.

[87] Rodríguez MR, Nuevo R, Chatterji S, Ayuso-Mateos JL (2012). Definitions and factors associated with subthreshold depressive conditions: a systematic review. BMC Psychiatry.

[88] Fergusson DM, Horwood LJ, Ridder EM, Beautrais AL (2005). Subthreshold depression in adolescence and mental health outcomes in adulthood. Arch Gen Psychiatry.

[89] Trivedi MH, Rush AJ, Wisniewski SR, Nierenberg AA, Warden D, Ritz L, Norquist G, Howland RH, Lebowitz B, McGrath PJ, Shores-Wilson K, Biggs MM, Balasubramani GK, Fava M, STAR*D Study Team (2006). Evaluation of outcomes with citalopram for depression using measurement-based care in STAR*D: implications for clinical practice. Am J Psychiatry.

[90] Donker T, Petrie K, Proudfoot J, Clarke J, Birch M-R, Christensen H (2013). Smartphones for smarter delivery of mental health programs: a systematic review. J Med Internet Res.

